# Inflammatory bowel disease and the risk of all caused or specific fracture: a meta-epidemiologic study

**DOI:** 10.3389/fendo.2026.1660702

**Published:** 2026-01-28

**Authors:** Weiren Wang, Hongfei Liu, Wei Wei, Guangzhi Zhou, Bohan Yu, Fumin Xue, Siwen Kang, Dongxu Tai

**Affiliations:** 1 The First Clinical College, Liaoning University of Traditional Chinese Medicine, Shenyang, Liaoning, China; 2 The Second Clinical College, Liaoning University of Traditional Chinese Medicine, Shenyang, Liaoning, China; 3 Second Department of Orthopaedics, The Second Affiliated Hospital of Liaoning University of Traditional Chinese Medicine, Shenyang, Liaoning, China; 4 Second Department of Orthopaedics, Affiliated Hospital of Liaoning University of Traditional Chinese Medicine, Shenyang, Liaoning, China

**Keywords:** cohort study, Crohn’s disease, fracture, inflammatory bowel disease, meta- epidemiologic study, ulcerative colitis

## Abstract

**Objective:**

To evaluate the overall and site-specific fracture risk in individuals with IBD through a meta-epidemiologic approach, synthesizing data from cohort studies and providing a comprehensive analysis of fracture risk at different anatomical sites.

**Methods:**

Following PRISMA 2020 guidelines, we systematically searched PubMed, Embase, and the Cochrane Library (inception to April 2025) for cohort studies reporting fracture risk in IBD patients. Eligible studies provided relative risk (RR) and 95% confidence interval(CI) for all-cause or site-specific fractures. Two reviewers independently screened records, extracted data, and assessed study quality using the Newcastle-Ottawa Scale (NOS). Random-effects meta-analysis, sensitivity/subgroup analyses, and publication bias assessment (funnel plots, Egger’s test) were performed.

**Result:**

Eleven cohort studies (4 prospective, 7 retrospective) from multiple countries were included, involving 2,102 to 54,591 IBD patients. NOS scores ranged from 5 to 8, indicating moderate to high study quality. Pooled analysis showed a 13% increased risk of all-cause fractures in IBD patients (RR = 1.13, 95% CI: 1.03–1.24; I²=70.8%, p<0.001). Subgroup analysis revealed higher fracture risks in Crohn’s disease (CD: RR = 1.23, 95% CI: 1.21–1.25) compared to ulcerative colitis (UC: RR = 1.16, 95% CI: 1.13–1.19). Site-specific risks were significantly higher for rib (RR = 1.24, 95% CI: 1.08–1.42; I²=0%, p=0.978), hip (RR = 1.39, 95% CI: 1.22–1.59; I²=54.2%, p=0.053), upper limb (RR = 1.46, 95% CI: 1.18–1.82; I²=94.6%, p<0.001), and lower limb fractures (RR = 1.60, 95% CI: 1.36–1.88; I²=75.4%, p<0.001). Sensitivity analyses confirmed the robustness of the results, and funnel plots/Egger’s test indicated no significant publication bias (p=0.612).

**Conclusion:**

IBD is associated with increased risks of all-cause and site-specific fractures, particularly in CD patients and lower limb fractures. These findings underscore the need for targeted bone health monitoring in IBD management.

**Systematic review registration:**

PROSPERO, identifier: CRD420251038879.

## 1 Introduction

Inflammatory bowel disease (IBD), comprising Crohn’s disease and ulcerative colitis, is a chronic inflammatory disorder of the gastrointestinal tract that has become a global health concern (1, 2). The global prevalence of IBD reached 6.8 million cases in 2017, with a subsequent decrease to 4.9 million in 2019 (3). IBD’s epidemiology progresses through stages: Emergence, Acceleration in Incidence, and Compounding Prevalence (4). While historically more prevalent in Western countries, IBD incidence is rising in developing and newly industrialized nations (5, 6). This global shift is attributed to factors such as Westernized diets, improved hygiene, and socioeconomic changes (5). The disease’s multifactorial pathogenesis involves genetic susceptibility, environmental triggers, and altered gut microbiome, necessitating further research for improved prevention and treatment strategies (1). Recent studies have recognized IBD as an independent risk factor for various health complications. Patients with IBD are at increased risk of cardiovascular diseases, atherosclerosis (7, 8), adverse pregnancy outcomes, oral health issues (9), and osteoporotic fractures (10). These findings highlight the need for comprehensive health management in IBD patients, including cardiovascular risk assessment and bone health monitoring (11, 12).

Fractures pose a significant health burden, leading to long-term disability, reduced quality of life, and increased healthcare costs (13). The association between IBD and increased fracture risk is complex and multifactorial. Contributing factors include corticosteroid use, common among IBD patients, which negatively affects bone mineral density (BMD) (14). Different pharmacologic agents exert distinct effects on bone metabolism. Biologics that target tumor necrosis factor-α (TNF-α), such as infliximab, can attenuate inflammation-induced bone loss, likely by normalizing the local cytokine milieu (15). At the mechanistic level, the RANK–RANKL–OPG axis is regarded as a central mediator IBD to bone loss. Numerous studies have demonstrated that chronic systemic inflammation in IBD leads to elevated levels of inflammatory cytokines, such as TNF-α and interleukin-6 (IL-6). These proinflammatory cytokines can promote osteoclastogenesis and increased bone resorption by altering the RANK–RANKL–OPG balance (for example, upregulating RANKL and/or reducing osteoprotegerin), thereby disrupting normal bone remodeling (16–18).Additionally, malabsorption of essential nutrients like calcium and vitamin D, due to intestinal inflammation or surgery, worsens bone fragility (19).

Despite these mechanistic insights, the magnitude of fracture risk in IBD remains contentious. Existing studies (19–29) report heterogeneous estimates for all-cause fractures and site-specific risks, likely due to variations in study design, follow-up duration, and adjustment for confounders such as steroid exposure or comorbidities. Up to now, the risks of all-cause fractures and specific fractures in IBD patients have not been uniformly concluded in different longitudinal population studies. Therefore, we conducted a meta-analysis based on cohort studies to further clarify the associations among them.

## 2 Methods

This meta-analysis was conducted following the PRISMA 2020 guidelines for systematic reviews (30). Our protocol was registered with PROSPERO (CRD420251038879).

### 2.1 Data sources

We searched PubMed, EMBASE, and the Cochrane Library from inception until April 25, 2025, with no restrictions. The subject terms (Emtree in Embase, MeSH in PubMed) and corresponding keywords were used. Search terms included those related to inflammatory bowel diseases, fractures and its variants. Search terms related to inflammatory bowel disease (IBD), fractures, and related variants were used. We also reviewed reference lists of retrieved studies and previous meta-analyses to identify additional eligible studies. Detailed search strategies for each database are provided in **Supplementary Tables 1**-**3**.

### 2.2 Study selection

Initial records were imported into NoteExpress reference management software, and duplicates were removed. Two reviewers (Hongfei Liu and Wei Wei) independently screened titles and abstracts to exclude irrelevant studies, classifying the remaining records as eligible, ineligible, or uncertain. Full texts of uncertain records were reviewed for eligibility. Discrepancies were resolved through group discussion.

### 2.3 Eligibility criteria

Studies were included if they met the following criteria: (a) Outcome: all-cause fractures or fractures at specific sites. (b) Exposure: confirmed diagnosis or medical history of IBD. (c) Control: healthy individuals or non-IBD patients. (d) Effect estimates: hazard ratio (HR), relative risk (RR), or odds ratio (OR) with 95% confidence intervals (CIs). Since IBD patients have a lower overall fracture risk, HR, RR, and OR were analyzed equivalently. (e) Study design: cohort studies (prospective or retrospective).

Studies were excluded if they: (a) were conference abstracts or letters to the editor, (b) were duplicate publications, (c) combined osteoporosis data without providing independent fracture data, (d) focused solely on fracture risk factors.

### 2.4 Data extraction

A data extraction form was created in Excel (Microsoft Corporation, USA). Two reviewers (Weiren Wang and Guangzhi Zhou) independently extracted the following data from eligible studies: first author, publication year, country, study type, research period, IBD classification, sample size, fracture incidence, follow-up time, and adjusted confounders. Extracted data were cross-checked, and disagreements were resolved through discussion.

### 2.5 Study quality

The quality of cohort studies was assessed using the Newcastle-Ottawa Quality Assessment Scale (NOS) (available at: http://www.ohri.ca/programs/clinical_epidemiology/oxford.asp). The NOS evaluates selection, comparability, and outcomes, with scores ranging from 0 to 9 stars. Studies with scores of 7 or higher, 4-6, and 0–3 were categorized as high, moderate, and low quality, respectively.

### 2.6 Data synthesis

Data analysis was performed using Stata software (version 14). Given the anticipated clinical, methodological, and statistical heterogeneity, a random-effects model was employed to enhance the robustness and interpretability of the results (31, 32). Sensitivity analyses were conducted to assess the stability of the overall findings and identify sources of heterogeneity. Subgroup analyses were performed based on fracture sites, study types, and regions to explore fracture risk across different IBD groups. Publication bias was evaluated using funnel plots and Egger’s regression test.

## 3 Results

### 3.1 Study selection

**Figure 1:**
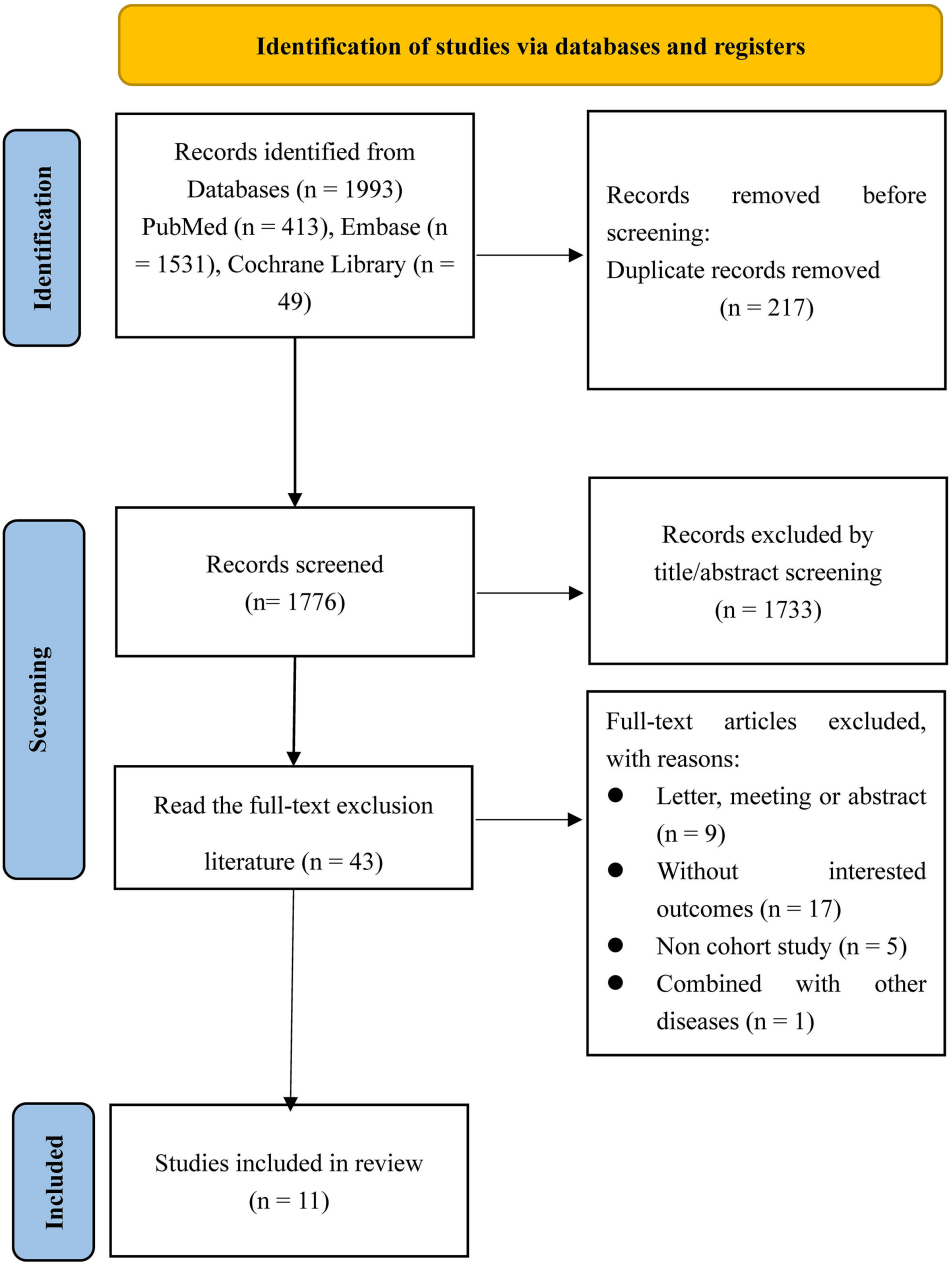
Flowchart of study selection.

A total of 1993 records were identified from databases (PubMed: 413, Embase: 1531, Cochrane Library: 49). After removing 217 duplicates, 1776 records were screened by title and abstract, resulting in 1733 exclusions. Full-text articles were then assessed, with 43 studies excluded for reasons including being a letter, meeting abstract, lack of relevant outcomes, non-cohort study design, or combining with other diseases. Ultimately, 11 studies (19–29) were included in this meta-analysis (**Figure 1**).

### 3.2 Studies characteristics

The included studies in this meta-analysis were predominantly cohort studies, with both prospective (n=4) and retrospective (n=7) designs, conducted across multiple countries including the USA, Denmark, Canada, Sweden, Korea, China, and the UK. The studies spanned enrollment periods from 1964 to 2018, with sample sizes ranging from 2,102 to 54,591 individuals diagnosed with IBD, including CD and UC. Fracture outcomes were diagnosed through medical records, radiologist reports, or health registries, with fracture types including hip, vertebral, radius/ulna, femur, spine, and others. Most studies adjusted for key confounders such as age, sex, steroid use, comorbidities, and disease duration, while some also considered histological inflammation/remission status and other demographic factors (**Table 1**).

**Table 1:** Basic characteristics of included studies.

Author	Year	Country	Study type	Data source	Enroll-period	IBD diagnostic criteria	IBD number	Fracture diagnostic criteria	Fracture number	Confounders adjusted
Mårild	2024	Sweden	Prospective Cohort study	National health care registers in Sweden	1990-2016	ICD or SNOMED^2^	IBD overall (54,591)	ICD	All fracture (1,480)	Age at index date (i.e. the start of the exposure period), sex, calendar year, education level, country of birth, disease duration, any history of inpatient IBD care, IBD-related surgery and the Charlson comorbidity index.
							Histological inflammation (43,449)			
							Histological remission (23,814)		Hip (123)	
Choi	2023	Korea	Prospective Cohort study	NHIS database	2008-2018	ICD-10 and RID registration system (V code)	IBD overall (33,778)	ICD-10	Vertebral (559)	Age, sex, and long-term steroid use.
							UC (24,370)			
							CD (9,408)			
Ahn	2022	Korea	Retrospective Cohort study	KNHI	2007-2016	KCD-6 and ICD-10	IBD overall (18,228)	ICD-10	All fracture (236)	The presence of hypertension, diabetes mellitus, dyslipidemia, stroke, osteoporosis, and use of systemic steroids.
							UC (12,011)		Vertebral (190)	
							CD (6,217)		Thoracic spine (85)	
									Lumbar spine (143)	
									Hip (55)	
									Femur neck (22)	
									Peri trochanter (20)	
									Other femur parts (13)	
Ludvigsson	2019	Sweden	Retrospective Cohort Study	Swedish National Patient Register	1964-2014	ICD	IBD overall (83,435)	ICD	Hip (2,491)	Sex, age, and place of residence.
							UC (50,162)		All fracture (12,931)	
							CD (26,763)			
							IBD unclassified (6,510)			
Tsai	2014	China	Retrospective Cohort Study	NHI	2000-2010	ICD-9	IBD overall (3,141)	ICD-9	Osteoporosis with pathologic fracture (11)	Age, sex and comorbidities.
Card	2004	UK	Retrospective Cohort Study	GPRD	1987-2001	Oxmis and Read coding	IBD overall (16,550)	Oxmis and Read coding	Hip (72)	Age, sex, current steroid use, cumulative steroid use, and opioid use.
							UC (8,301)			
							CD (5,960)			
Van Staa	2003	UK	Retrospective Cohort Study	GPRD	1987-1999	ICD-9	IBD overall (2,102)	ICD-9	All fracture (1,134)	Medications and illnesses associated with risk of fracture (as outlined in Patients and Methods) and smoking history and body mass index when known.
							UC (1,305)		Hip (106)	
							CD (725)		Vertebral (74)	
									Radius/ulna (253)	
Loftus	2003	USA	Retrospective Cohort Study	Medical records	1940-1993	NR	UC (273)	Radiologist’s report	Skull/face (4)	NR
									Hands/fingers (19)	
									Distal forearm (2)	
									Another arm (7)	
									Clavicle/scapula/sternum (5)	
									Ribs (10)	
									Thoracic/lumbar vertebrae (17)	
									Proximal femur (5)	
									Other leg (11)	
									Feet/toes (7)	
									Any site (60)	
Vestergaard	2002	Denmark	Cohort study	The National Patient Discharge Register	1983-1996	ICD-8	CD (7,072)	ICD-8 and ICD-10	All fractures (931)	NR
									Skull and jaws (28)	
									Spine, rib, and pelvis (66)	
							UC (8,323)		Upper arm (105)	
									Forearm (164)	
									Colles’ fracture (123)	
									Hand and finger (164)	
							Celiac disease (1,021)		Hip and femur (240)	
									Femoral neck (207)	
									Lower leg (164)	
									Foot (56)	
Loftus	2002	USA	Retrospective Cohort study	Medical records	1940-1993	NR	CD (238)	Radiologist’s report	Skull/face (2)	NR
									Hands/fingers (10)	
									Distal forearm (7)	
									Another arm (6)	
									Clavicle/scapula/sternum (3)	
									Ribs (5)	
									Thoracic/lumbar vertebrae (15)	
									Other vertebrae (2)	
									Pelvis (1)	
									Proximal femur (5)	
									Other leg (8)	
									Feet/toes (20)	
									Osteoporotic (hip, wrist, spine) (20)	
									All fractures (44)	
Bernstein	2000	Canada	Cohort study	University of Manitoba IBD Database	1984-1997	ICD-9	CD (184)	ICD-9	All fractures (405)	NR
							UC (221)			
							IBD overall (405)			
							CD (53)			
							UC (54)			

ICD, International Classification of Disease; NHIS, Systematized Nomenclature of Medicine; NHIS, National Healthcare Insurance Service; RID, Rare Intractable Diseases; KNHI, The Korean National Health Insurance; KCD, Korean Classification of Diseases; GPRD, The General Practice Research Database.

### 3.3 Quality of included studies

The total score for each study ranged from 5 to 8 stars, reflecting moderate to high quality. Most studies (n=8) scored 7 or 8 stars, indicating robust methodological quality, particularly in the domains of selection and outcome assessment. However, the comparability domain showed some variability, with most studies receiving 1 to 2 stars. The overall results suggest that the included studies generally met the NOS criteria, although there were slight differences in the comparability of study populations. The detailed assessment scores are presented in **Table 2**.

**Table 2:** The quality of included studies.

Study	Year	Selection	Comparability	Outcome	Total
Mårild	2024	***	**	***	8
Choi	2023	**	*	**	5
Ahn	2022	****	**	**	8
Ludvigsson	2019	***	**	***	8
Tsai	2014	***	**	**	7
Card	2004	***	**	**	7
Van Staa	2003	****	*	**	7
Loftus	2003	***	**	***	8
Vestergaard	2002	***	**	***	8
Loftus	2002	***	**	***	8
Bernstein	2000	***	**	**	7

### 3.4 IBD and the risk of all-cause fractures

**Figure 2:**
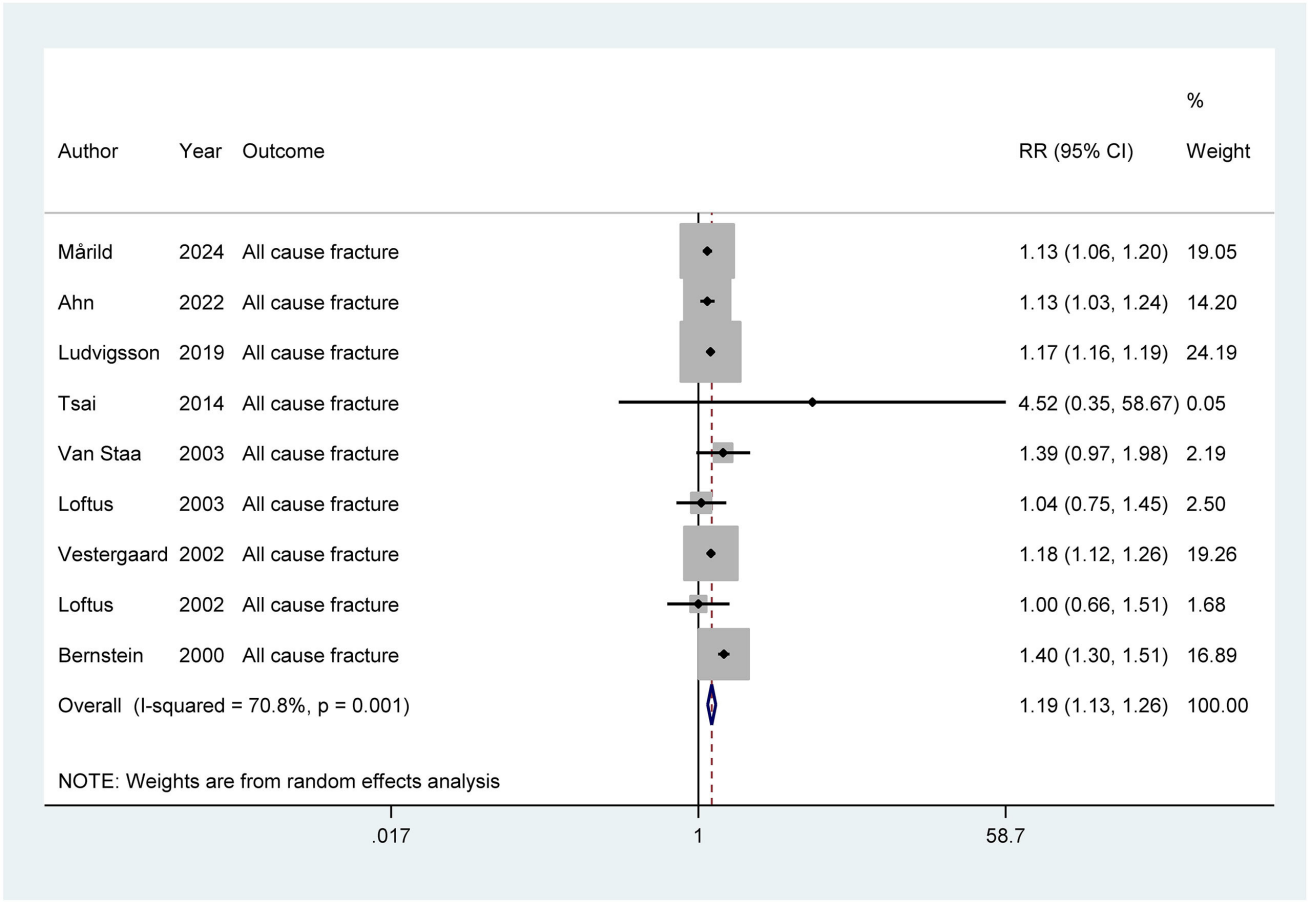
Meta-analysis of IBD and the risk of all-cause fractures.

**Figure 3:**
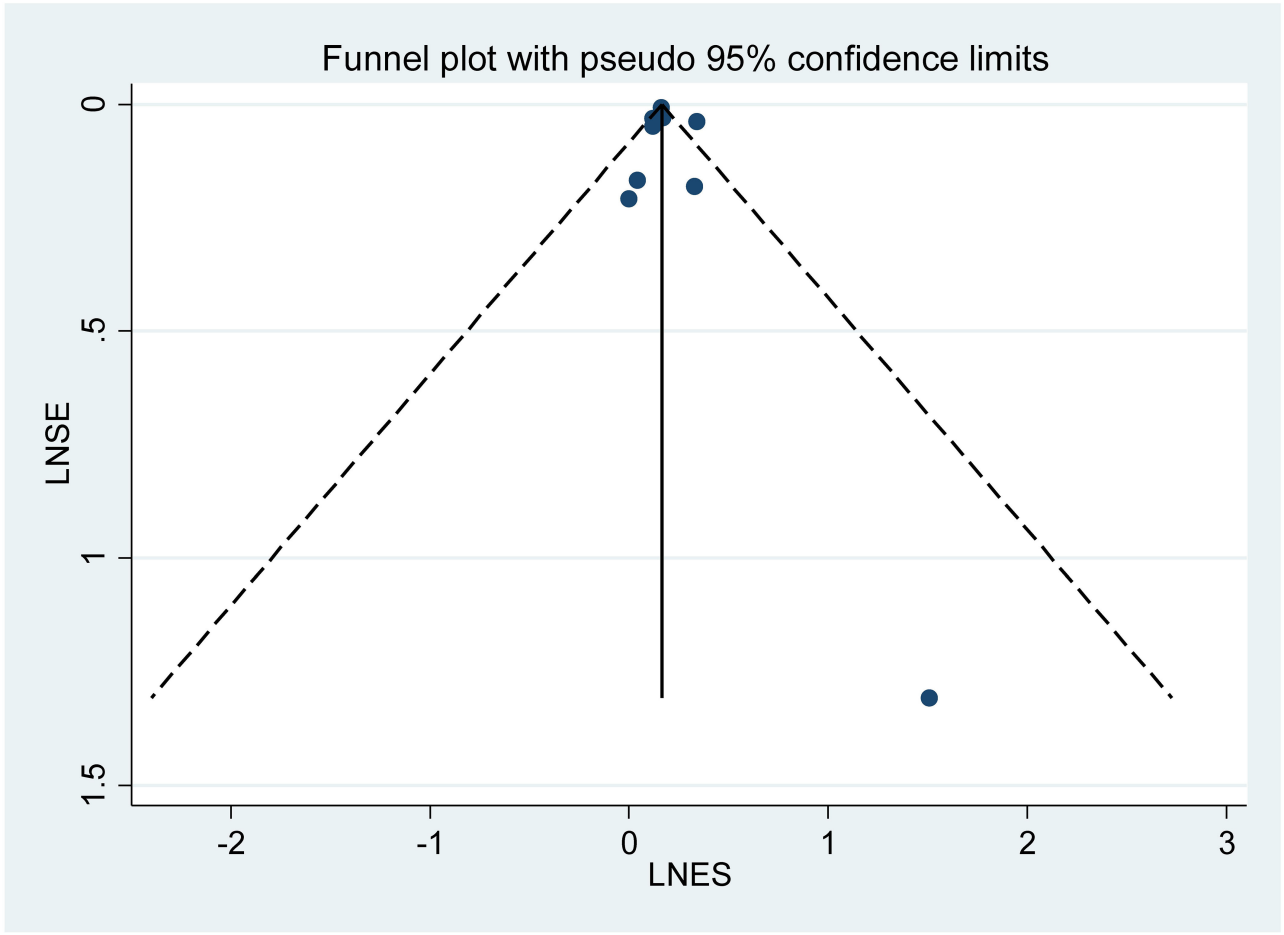
Funnel plot for the risk of all-cause fractures in IBD.

A total of 9 studies were included, with the pooled RR for all-cause fractures in individuals with IBD being 1.13 (95% CI: 1.03–1.24, *P* = 0.003, τ²=0.0056). The overall heterogeneity, as indicated by I², was 70.8%, suggesting a high level of variability across the studies (**Figure 2**). The sensitivity analysis assessed the stability of the meta-analysis results by evaluating the influence of individual studies on the overall effect size. Exclusion of any single study did not substantially alter the pooled effect estimates, indicating that the overall findings are robust and not overly dependent on any one study (**Supplementary Figure 1**). The funnel plot visually indicates the presence of publication bias, which was formally assessed using Egger’s test. The Egger’s test value of 0.612 suggests no significant publication bias, as the plot appears symmetric (**Figure 3**).

We conducted subgroup analyses based on different pathological types of IBD and the risk of all-cause fractures (**Table 3**). The pooled RR for all-cause fractures was higher in patients with CD (1.23, 95% CI: 1.21–1.25) compared to those with UC (1.16, 95% CI: 1.13–1.19). The overall IBD group showed a similar risk (RR: 1.13, 95% CI: 1.03–1.24).

**Table 3:** Subgroup analysis for different IBD type and all cause fracture.

Type	No. of studies	RR (95% CI)	*P* value	I² (%)
Overall IBD	9	1.13 (1.03, 1.24)	0.001	70.8
CD	6	1.23 (1.21, 1.25)	0.001	84.6
UC	6	1.16 (1.13, 1.19)	0.001	98.2

### 3.5 IBD and the risk of specific fracture

We analyze the risks of specific fractures at different anatomical sites, focusing particularly on rib, hip, upper limb, and lower limb fractures (**Table 4**). This meta-analysis investigates the risk of rib fractures in patients with IBD across 6 studies. The pooled relative risk (RR) for rib fractures in IBD patients was 1.24 (95% CI: 1.08–1.42), indicating a modest increase in fracture risk compared to the general population. The studies exhibited low heterogeneity (I² = 0%, p = 0.978), suggesting that the findings were consistent across the included studies (**Supplementary Figure 2**).

**Table 4:** The risk of specific fracture in IBD.

Fracture type	No. of studies	RR (95% CI)	P value	I² (%)
Rib Fractures	6	1.24 (1.08, 1.42)	0.003	0.0
Hip Fractures	7	1.39 (1.22, 1.59)	0.001	54.2
Upper Limb Fractures	5	1.46 (1.18, 1.82)	0.001	94.6
Lower Limb Fractures	6	1.60 (1.36, 1.88)	0.001	75.4

We also assessed the risk of hip fractures in IBD patients, incorporating data from 7 studies. The pooled RR for hip fractures was 1.39 (95% CI: 1.22–1.59), reflecting a significant increase in fracture risk in this population. The overall heterogeneity was moderate (I² = 54.2%, p = 0.053), indicating some variability in study outcomes (**Supplementary Figure 3**).

Next, we examined the risk of upper limb fractures in IBD patients, combining data from 5 studies. The pooled RR for upper limb fractures was 1.46 (95% CI: 1.18–1.82), suggesting a moderate but statistically significant increased risk of fractures in the upper limbs. The heterogeneity was high (I² = 94.6%, p = 0.000), indicating considerable variability across studies, particularly in how different study populations report or define upper limb fractures (**Supplementary Figure 4**).

Lastly, we evaluated the risk of lower limb fractures in IBD patients, combining 6 studies. The pooled RR for lower limb fractures was 1.60 (95% CI: 1.36–1.88), indicating a notably higher risk for IBD patients. The studies showed high heterogeneity (I² = 75.4%, p = 0.000), suggesting substantial differences in study designs and populations (**Supplementary Figure 5**).

## 4 Discussion

### 4.1 Main findings

This meta-analysis of 11 cohort studies provides robust evidence that patients with IBD face a significantly increased risk of both all-cause and site-specific fractures. Our findings demonstrate a 13% elevated risk of all-cause fractures (RR = 1.13, 95% CI: 1.03–1.24, τ²=0.0056), with notable heterogeneity (I²=70.8%, p<0.001). Subgroup analyses further revealed distinct risk patterns: CD patients exhibited higher fracture risk compared to UC (RR = 1.23 vs. 1.16), and lower limb fractures posed the greatest site-specific risk (RR = 1.60, 95% CI: 1.36–1.88). These results align with prior studies linking IBD to skeletal complications but extend existing knowledge by quantifying site-specific risks and addressing methodological gaps in previous meta-analyses.

### 4.2 Comparison with previous meta-analyses

Our meta-analysis corroborates and expands upon previous investigations into fracture risk in IBD. Consistent with prior studies (10), we observed a significant elevation in all-cause fracture risk (RR = 1.13 vs. RR = 1.20 in Bernstein et al.), reinforcing IBD as an independent risk factor for skeletal complications. Similar to Hidalgo et al. (33), our analysis identified CD as a higher-risk subtype compared to UC (RR = 1.23 vs. 1.16), likely due to CD’s more extensive intestinal involvement and malabsorption sequelae. However, our study diverges methodologically by restricting inclusion to cohort studies (n=11), enhancing causal inference, whereas prior meta-analyses often combined case-control and cohort designs, introducing susceptibility bias. Notably, Szafors et al. (34) reported comparable hip fracture risks (RR = 1.39 vs. RR = 1.35 in their analysis), but our findings uniquely quantified lower limb fractures as the highest site-specific risk (RR = 1.60), a novel observation not previously emphasized. Additionally, our stratified analyses revealed minimal heterogeneity for rib fractures (I²=0%), contrasting with the high heterogeneity in prior syntheses (I²=67–89%), potentially due to stricter confounder adjustments (e.g., steroid dose, disease duration). These advancements underscore the clinical imperative for site-specific fracture prevention strategies, particularly targeting CD patients and lower limb injuries, while addressing methodological inconsistencies in earlier reviews.

### 4.3 The related mechanisms of IBD and increased risk of fractures

The elevated fracture risk in patients with IBD is mediated by multifactorial mechanisms rooted in the pathophysiology of IBD and its management. First, chronic systemic inflammation drives bone loss through cytokine-mediated pathways. Pro-inflammatory cytokines such as TNF-α and IL-6 directly promote osteoclastogenesis while suppressing osteoblast activity, disrupting bone remodeling (10). The RANK/RANKL/OPG pathway also plays a pivotal role, chronic IBD inflammation upregulates RANKL, inhibiting OPG and favoring osteoclast differentiation. Second, prolonged corticosteroid use, a cornerstone of IBD therapy, induces osteoblast apoptosis, reduces intestinal calcium absorption, and suppresses adrenal androgen synthesis, collectively accelerating BMD loss (14), whereas emerging biologics and immunomodulators may offer protective effects by reducing systemic cytokine burdens, potentially lowering fracture risks in treated patients (15). Third, nutritional deficiencies secondary to intestinal inflammation or resection impair calcium and vitamin D absorption, exacerbating skeletal fragility (19). Vitamin D deficiency, prevalent in IBD populations, not only compromises calcium homeostasis but also exacerbates dysbiosis and systemic inflammation, further perpetuating bone resorption (35). Fourth, surgical interventions (e.g., small bowel resections in Crohn’s disease) reduce the absorptive surface area for nutrients critical to bone health, compounding fracture risk (36). Lastly, reduced physical activity due to IBD-related fatigue, pain, or disability contributes to lower limb muscle wasting and decreased mechanical loading on bones, predisposing to fragility fractures (37). These mechanisms synergistically amplify fracture susceptibility in IBD patients, particularly those with CD, who often experience more severe malabsorption and inflammation.

### 4.4 Limitations and clinical implications

This meta-analysis has several limitations. First, significant heterogeneity was observed across studies (e.g., I²=94.6% for upper limb fractures), likely due to variations in fracture definitions, diagnostic criteria (e.g., radiologist reports vs. ICD codes), and adjustment for confounders (e.g., inconsistent adjustment for steroid dose or disease duration). The high level of heterogeneity limits the robustness of our conclusions, and further research with consistent standards and comprehensive adjustment for confounding factors is needed to validate these findings. Potential sources of this heterogeneity include differences in fracture definitions (e.g., reliance on ICD codes in large administrative databases versus radiology-confirmed fractures in smaller cohorts), varying follow-up durations (ranging from 7 to 50 years across studies), and inconsistencies in baseline patient characteristics such as age, sex distribution, and disease severity. Additionally, adjustment for key confounders varied: while some studies controlled for cumulative steroid exposure—a major contributor to bone loss—others did not, and comorbidities like osteoporosis or vitamin D deficiency were inconsistently accounted for. Due to the limited number of studies and incomplete reporting of covariates at the study level, meta-regression was not feasible. we performed additional subgroup analyses by study design. These findings emphasize the need for standardized reporting in future cohort studies. Second, this study conducted a meta-analysis based on observational cohort studies spanning several decades, from different countries, and with varying methodological definitions. Such a design inevitably introduces limitations in controlling for confounding factors and making causal inferences. Therefore, the findings of this study provide evidence of associations rather than definitive causal relationships. To minimize this limitation, we prioritized the inclusion of high-quality cohort studies and performed sensitivity analyses by excluding lower-quality studies to assess the robustness and consistency of the results. Third, most included cohorts originate from Western (e.g., Europe, USA) and East Asian (e.g., Korea, China) populations, limiting direct generalizability to underrepresented regions such as Africa, South America, and the Middle East, where IBD epidemiology and access to bone health screening may differ. Future studies should prioritize diverse global cohorts to address these gaps and validate findings across varied socioeconomic and genetic contexts. Fourth, publication bias could not be fully ruled out despite nonsignificant Egger’s test results (p=0.612), as non-English studies were excluded. Fifth, the reliance on aggregated data precluded detailed analyses of fracture severity, recurrence, or the impact of specific IBD therapies (e.g., biologics) on bone health. Lastly, while the Newcastle-Ottawa Scale indicated moderate-to-high study quality, variability in comparability domains (e.g., differences in control group selection) may introduce bias. These limitations highlight the need for standardized fracture reporting and prospective studies with granular data to refine risk stratification. Our findings underscore the need for proactive bone health management in IBD care. Given the elevated fracture risks identified, it is crucial for clinicians to prioritize bone health monitoring in IBD patients, particularly those with CD, who demonstrate a higher risk of fractures compared to patients with UC. Subgroups to prioritize for DXA screening include patients with long disease duration (>10 years), high cumulative steroid dose (>7.5 mg/day prednisone equivalent for ≥3 months), prior low-trauma fractures, or additional risk factors like postmenopausal status or vitamin D deficiency. Screening should commence at IBD diagnosis for high-risk patients, with follow-up every 1–2 years based on initial BMD and disease activity. A Danish inception cohort study (38) exemplifies a prospective bone-health surveillance strategy: routine DXA assessment of all newly diagnosed IBD patients can facilitate early detection of osteopenia/osteoporosis and, when combined with appropriate therapeutic interventions, may help mitigate bone loss and reduce long-term fracture risk. For patients with diagnosed osteoporosis or very high fracture risk, initiation of bone-sparing pharmacotherapy (e.g. bisphosphonates, denosumab) is recommended according to standard osteoporosis treatment guidelines. These measures together can help mitigate fracture risk in the IBD population.

In addition to DXA screening, early identification of bone health issues allows for timely interventions. For example, calcium and vitamin D supplementation is essential for improving bone health in IBD patients, as these nutrients are often malabsorbed due to intestinal inflammation or surgical resection. Vitamin D plays a critical role not only in calcium absorption but also in modulating immune responses, which is particularly relevant in inflammatory conditions like IBD. Therefore, maintaining adequate levels of calcium and vitamin D should be a cornerstone of IBD management, particularly in patients at high risk of osteoporosis and fractures.

Furthermore, weight−bearing exercises, such as walking, jogging, or resistance training, can help stimulate bone formation and improve muscle strength, which is crucial for preventing falls and fractures. These exercises should be encouraged as part of a comprehensive bone health strategy, tailored to the individual’s physical capabilities and IBD disease activity.

Finally, for patients at significant fracture risk, clinicians should consider the judicious use of bone−sparing agents, such as bisphosphonates, denosumab, or selective estrogen receptor modulators. These medications can help prevent bone loss and reduce the risk of fractures, particularly in patients with severe osteoporosis or those who have been on long−term corticosteroid therapy. However, the use of these agents should be carefully monitored, taking into account potential side effects and the patient’s overall treatment plan for IBD.

In summary, incorporating proactive bone health management into routine IBD care is essential to reduce fracture−related morbidity and improve patient outcomes. By adopting a multidisciplinary approach that includes screening, supplementation, exercise, and appropriate pharmacologic interventions, clinicians can mitigate the risk of fractures and enhance the quality of life for IBD patients. At the same time, more refined stratified analyses are of high value. Future studies should report fracture outcomes using standardized, comparable definitions stratified by key clinical IBD characteristics (e.g., disease duration and activity), cumulative corticosteroid exposure, and specific treatment modalities; such harmonized reporting would enable reliable subgroup analyses to identify patient groups that may truly benefit from targeted interventions.

## 5 Conclusion

IBD is associated with an elevated risk of both all-cause and site-specific fractures, particularly among patients with CD and those experiencing lower limb fractures. This meta-analysis, rigorously conducted using cohort studies, underscores the importance of integrating bone health monitoring into routine IBD management to mitigate fracture-related morbidity. Future research should focus on elucidating underlying mechanisms and developing targeted prevention strategies to address this critical aspect of IBD care.

## Data Availability

The original contributions presented in the study are included in the article/**Supplementary Material**. Further inquiries can be directed to the corresponding authors.
