# Supplementary material for: Inflammatory bowel disease and the risk of all caused or specific fracture: a meta-epidemiologic study
Source: Front Endocrinol (Lausanne). 2026 Jan 28;17:1660702. doi: 10.3389/fendo.2026.1660702 (PMC12890657; doi:10.3389/fendo.2026.1660702)
Supplement: Supplementary file 1 [file DataSheet1.docx]

Supplementary Material

**The retrieval strategies and retrieval results of each database are shown in Supplementary Tables 1-3**.

**1.Supplementary Table 1: PubMed** **2025.04.25**

| No. | Query | Results |
| --- | --- | --- |
| 1 | "Inflammatory Bowel Diseases"[Mesh] | 105,530 |
| 2 | Inflammatory Bowel Disease*[Title/Abstract] | 74,814 |
| 3 | ("Inflammatory Bowel Diseases"[Mesh]) OR (Inflammatory Bowel Disease*[Title/Abstract]) | 135,408 |
| 4 | "Fractures, Bone"[Mesh] | 220,544 |
| 5 | (Fracture*[Title/Abstract]) OR (Broken Bone*[Title/Abstract]) | 343,896 |
| 6 | ("Fractures, Bone"[Mesh]) OR ((Fracture*[Title/Abstract]) OR (Broken Bone*[Title/Abstract])) | 386,854 |
| 7 | (("Inflammatory Bowel Diseases"[Mesh]) OR (Inflammatory Bowel Disease*[Title/Abstract])) AND (("Fractures, Bone"[Mesh]) OR ((Fracture*[Title/Abstract]) OR (Broken Bone*[Title/Abstract]))) | 413 |

**2.Supplementary Table 2 Embase 2025.04.25**

| No. | Query | Results |
| --- | --- | --- |
| #1 | 'inflammatory bowel disease'/exp | 233439 |
| #2 | 'inflammatory bowel disease*':ab,ti | 119589 |
| #3 | #1 OR #2 | 260076 |
| #4 | 'fracture'/exp | 426041 |
| #5 | fracture*:ab,ti OR 'broken bone*':ab,ti | 415384 |
| #6 | #4 OR #5 | 532636 |
| #7 | #3 AND #6 | 1531 |

**3.Supplementary Table 3 Cochran Library 2025.04.25**

| No. | Query | Results |
| --- | --- | --- |
| 1 | MeSH descriptor: [Inflammatory Bowel Diseases] explode all trees | 5002 |
| 2 | (Inflammatory Bowel Disease*):ti,ab,kw (Word variations have been searched) | 5343 |
| 3 | #1 OR #2 | 8625 |
| 4 | MeSH descriptor: [Fractures, Bone] explode all trees | 9582 |
| 5 | (Fracture*):ti,ab,kw OR (Broken Bone*):ti,ab,kw (Word variations have been searched) | 31506 |
| 6 | #4 OR #5 | 31548 |
| 7 | #3 AND #6 | 49 |

**Supplementary Figure 1: Sensitivity analysis for IBD and the risk of all-cause fractures**

**Supplementary Figure 2: Risk of Rib Fractures in IBD Patients**

**Supplementary Figure 3 Risk of Hip Fractures in IBD Patients**

**Supplementary Figure 4 Risk of Upper Limb Bone Fractures in IBD Patients**

**Supplementary Figure 5 Risk of Lower Limb Bone Fractures in IBD Patients**
